# Response of exercise‐onset vasodilator kinetics to L‐citrulline supplementation during different phases of the menstrual cycle

**DOI:** 10.14814/phy2.14536

**Published:** 2020-08-09

**Authors:** Joaquin U. Gonzales, Stephen M. Fischer, Arun Maharaj, Heather Vellers, Todd Anderson, Adcharee Karnjanapiboonwong, Seenivasan Subbiah, J. M. Kellawan, Arturo Figueroa

**Affiliations:** ^1^ Department of Kinesiology and Sport Management Texas Tech University Lubbock TX USA; ^2^ Department of Environmental Toxicology Texas Tech University Lubbock TX USA; ^3^ Department of Health and Exercise Science University of Oklahoma Norman OK USA

**Keywords:** blood flow, l‐citrulline, menstrual cycle, vasodilator kinetics

## Abstract

The purpose of this study was to determine whether L‐citrulline (CIT) supplementation during the follicular and luteal phases of the menstrual cycle would present differential effects on vasodilator kinetics in dynamically contracting muscle. Twenty‐four women were studied during the follicular (day 15 after onset of menses, *n* = 13) or the luteal phase (day 25 after onset of menses, *n* = 11). Supplementation with CIT (6g/day) or placebo occurred 7‐days prior to testing in a crossover design across two menstrual cycles. Forearm vascular conductance (FVC) was calculated from blood flow and mean arterial pressure measured continuously during handgrip exercise performed at 10% maximal grip strength. FVC was calculated for each duty cycle (contract:relax, 1:2s) and expressed as a change from baseline (ΔFVC) before being fit with a monoexponential model. Amplitude of the ΔFVC response and the number of duty cycles for ΔFVC to reach 63% of steady‐state amplitude (τΔFVC) were derived from the model. Analysis of variance showed no difference in the amplitude of ΔFVC between CIT and placebo (*p* = .45) or between menstrual cycle phases (*p* = .11). Additionally, τΔFVC was not different (*p* = .35) between CIT and placebo in women tested during the follicular (6 ± 3 versus 5 ± 3 duty cycles) or luteal phase (9 ± 1 versus 8 ± 1 duty cycles) although τΔFVC was found to be slower for women tested during the luteal as compared to the follicular phase (8 ± 4 versus 5 ± 3 duty cycles, *p* = .02). These results indicate that exercise‐onset vasodilator kinetics is unaltered with CIT supplementation in young healthy women irrespective of menstrual cycle phase.

## Graphical abstract

Exercise‐onset vasodilator kinetics was unaltered with L‐citrulline supplementation in young women tested during the follicular and luteal phase indicating that fluctuations in estrogen and progesterone do not influence the ability of L‐citrulline to enhance substrate availability for nitric oxide production. Vasodilator kinetics were faster during the follicular than luteal phase consistent with the notion that peripheral vascular reactivity may vary across the menstrual cycle.

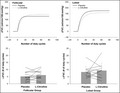

## 1 INTRODUCTION

It is well established that short‐term oral supplementation with L‐citrulline (CIT) significantly increases plasma levels of L‐arginine (ARG) (Bailey et al., 2015; Kim et al., 2015; Schwedhelm et al., 2008). Increased plasma ARG following oral CIT supplementation has been reported even in healthy young adults following a single dose (Kim et al., 2015) or after 7‐days (Bailey et al., 2015) of supplementation. When consumed orally, CIT is metabolized in the kidney by the enzymes argininosuccinate synthase and argininosuccinate lyase in a partial urea cycle to form ARG (Bahri et al., 2013). De novo synthesized ARG enters the systemic circulation after leaving the kidney via the renal vein (van de Poll, Soeters, Deutz, Fearon, & Dejong, 2004). Pharmacokinetic data show plasma levels of ARG increase in a dose‐dependent fashion after 7 days of CIT supplementation, with a 3‐g dose taken twice per day (6g per day) being effective at increasing ARG bioavailability (Schwedhelm et al., 2008). Importantly, circulating ARG can enter the endothelium where it serves as substrate for nitric oxide synthase (NOS), producing the vasodilator molecule nitric oxide (Kim et al., 2015).

The vasodilatory response to exercise is partially mediated by the ability of nitric oxide to reduce vascular tone. Prior work demonstrates that inhibition of NOS with *N* (G)‐monomethyl‐L‐arginine (L‐NMMA) reduces the forearm vascular conductance (FVC) response to dynamic handgrip exercise as observed by a prolongation of time to reach steady state (Casey, Mohamed, & Joyner, 2013). This finding has been replicated by other studies that have included young and older women (Casey, Ranadive, & Joyner, 2015; Kellawan et al., 2017), although discrepant results exist that show no effect of NOS inhibition on vasodilator kinetics at the onset of exercise (Shoemaker, Halliwill, Hughson, & Joyner, 1997). In addition, inhibition of NOS results in a significant absolute reduction in steady‐state FVC during exercise in young women (Kellawan et al., 2015), indicating that nitric oxide also contributes to the regulation of the overall muscular vasodilatory response to handgrip exercise. However, the contribution of nitric oxide to exercise‐induced vasodilation may differ across the normal menstrual cycle as estrogen and progesterone fluctuate. For instance, elevated brachial artery flow‐mediated dilation is reported during the follicular phase when plasma levels of estrogen and markers of nitric oxide production are elevated in the blood (Adkisson et al., 2010; Schnabel et al., 2013) Interestingly, evidence also exist that progesterone may antagonize the positive effect of estradiol on brachial artery reactivity (Miner et al., 2011). Whether fluctuations in sex hormones across the menstrual cycle influence the ability of CIT supplementation to enhance nitric oxide bioactivity has yet to be investigated.

Coupling of ARG to endothelial NOS is modulated by the female sex hormones estrogen and progesterone. ARG moves from the plasma to membrane‐bound endothelial NOS by the cationic amino acid transporter‐1 (CAT‐1) (McDonald, Zharikov, Block, & Kilberg, 1997). The CAT‐1 transporter is inhibited through several mechanisms, including posttranslational modulation by phosphorylated extracellular signal‐regulated kinase (ERK) and protein kinase Cɑ (PKCɑ). Bentur et al. (2015) demonstrated in cultured human endothelial cells that estrogen decreases phosphorylation of ERK resulting in elevated ARG transport through CAT‐1, while progesterone increases phosphorylated ERK and PKCɑ resulting in impaired ARG transport. In women, circulating ARG levels vary across the normal menstrual cycle with ARG decreasing during the luteal phase (Faustmann et al., 2018) and showing an inverse association with progesterone levels in blood (Valtonen et al., 2010). The cause of reduced plasma ARG levels is unknown, but could have an influence on ARG bioavailability for nitric oxide production. To date, no study has examined the effect of increasing plasma levels of ARG with CIT supplementation during the follicular and luteal phases, but these results may have implications for improving functional hyperemia for women in sport or with cardiovascular disease.

This study aimed to determine the ability of CIT supplementation to alter the vasodilator response to dynamic handgrip exercise during the follicular or luteal phase of the normal menstrual cycle. We hypothesized that CIT supplementation would enhance the FVC response to exercise during the follicular phase, but that this response would be absent during the luteal phase based on the inhibitory effect of progesterone on ARG transport shown by others (Bentur et al., 2015).

## 2 METHODS

### 2.1 Ethical approval

All participants provided written informed consent prior to data collection. The Human Research Protection Program at Texas Tech University provided ethics approval for this study (#IRB2018‐751). This study and its procedures conformed to standards set by the latest version of the Declaration of Helsinki, with the exception that the study is not registered in a publicly accessible database.

### 2.2 Participants

Twenty‐four women (21–30 years) participated in this study. Participants had no history of cardiovascular disease, renal disease, and were not taking medication for high blood pressure or any form of medical birth control, including oral contraceptives. Participants were nonsmokers, had a body mass index < 30 kg/m^2^, fasting blood glucose < 125 mg/dL based on finger prick (AccuChek Active, Roche Diagnostics), and reported having regular menstrual cycles as demonstrated by being eumenorrheic for each of the preceding three months.

### 2.3 Study design

**Figure 1:**
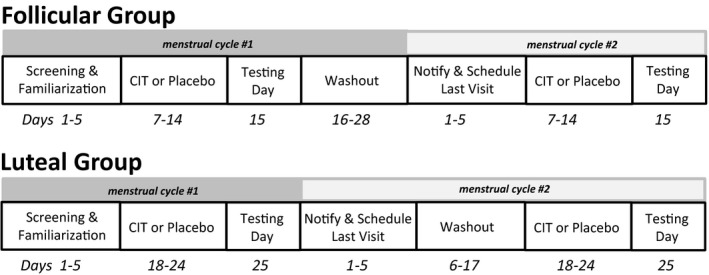
Crossover study design. Women were randomized into the follicular phase group or luteal phase group. The follicular phase group was supplemented with L‐citrulline (CIT) or placebo during days 7–14 of the normal menstrual cycle, while women in the luteal phase group took CIT or placebo capsules during days 18–24 of the normal menstrual cycle. Both groups of women crossed over to taking CIT or placebo during a second menstrual cycle. The order of which capsules were taken first was randomized between subjects within each group

This study was a randomized, double‐blind, crossover study involving CIT (NOW Foods, Bloomingdale, IL) and maltodextrin (NOW Foods) that was given as a placebo control. As shown in Figure 1, CIT (6 g/day, instructed to take half at lunch and half at dinner) and placebo were taken orally for 7‐days across two consecutive menstrual cycles. The order that women took CIT and placebo was randomized. Women placed in the follicular phase group took CIT or placebo on days 7–14 from the start of menses, while women in the luteal group took CIT or placebo on days 18–24 from the start of menses. The rationale for this design is that women would be consuming CIT (i.e., increasing ARG bioavailability) during a 7‐day period when estradiol or progesterone likely peaked during their respective phases. The last dose of CIT or placebo was consumed the night before the study visit, consistent with our past work (Ashley, Kim, & Gonzales, 2018; Figueroa, Trivino, Sanchez‐Gonzalez, & Vicil, 2010; Gonzales, Raymond, Ashley, & Kim, 2017), which occurred on day 15 from the start of menses for the follicular group and day 25 from the start of menses for the luteal group. All visits were scheduled in the morning, and participants were instructed to arrive after refraining from food, caffeine, vitamins, and dietary supplements for at least 8 hr.

### 2.4 Experimental protocol

This study was comprised of three visits (Figure 1). The first visit was scheduled when women reported being in menses. This visit consisted of informed consent, medical history questionnaire, seated resting blood pressure (HEM‐907XL, Omron Healthcare, Lake Forest, IL), maximal grip strength testing, a step test to estimate cardiorespiratory fitness level, and familiarization of the exercise protocol. The second and third visits were the same in procedures and testing time, but differed based on whether CIT or placebo was taken prior to the visit. Upon arriving for these last two visits, blood was collected to measure female sex hormones and plasma ARG levels. Lastly, women performed rhythmic dynamic handgrip exercise as forearm blood flow was measured using Doppler ultrasound.

### 2.5 Fitness test

The Queen's College step test was used to estimate maximal oxygen uptake (McArdle, Katch, Pechar, Jacobson, & Ruck, 1972). The test requires women to step upon a 16.2 inch box in a bipedal manner following an up‐up‐down‐down pattern at a rate of 22 steps per minute. To accomplish this rate, an audio metronome was set to 88 beeps per minute. Heart rate was measured by palpation at the left radial artery for 15s immediately after women completed the 3min test. Maximal oxygen uptake (VO_2max_) was then estimated using the equation 65.81 – (0.1847 x heart rate).

### 2.6 Handgrip exercise

Participants rested in a supine position for at least 10 min before performing dynamic handgrip exercise for 5 min using their right hand. The right forearm was secured to a table with a Velcro strap and foam pad to minimize movement of the arm during exercise. Contraction rate was set using a metronome sounding at 40 beeps per minute. Participants were instructed to contract quickly upon one beep (distance traveled was ~ 5cm) and to return to the resting position before skipping the other beep. This resulted in 20 contractions per minute with a contraction:relaxation cycle of ~ 1:2s. Workload was set at 10% maximal voluntary contraction force and did not vary between visits. Maximal force was determined using a grip strength dynamometer (model 5001, Takei Scientific Instruments, Niigata City, Japan), and was considered the peak force measured after three attempts with 1‐min rest between trials. This workload was selected as pilot work showed higher intensities produced early fatigue, and past research has confirmed that blood flow responses to exercise at this intensity are reduced with inhibition of NOS with L‐NMMA in young adults (Casey et al., 2015). Participants were given visual feedback in the form of displacement curves that showed distance traveled during each concentric contraction that was recorded by a potentiometer incorporated into the custom‐built handgrip ergometer. Participants were encouraged to aim for consistency in the shape and frequency of displacement curves during exercise.

### 2.7 Forearm blood flow and conductance

Diameter was measured in the right brachial artery using Doppler ultrasound (Vivid 7, GE Healthcare, Waukesha, WI) with a 5–13 MHz linear transducer probe at rest and during the last 30s of handgrip exercise. Blood velocity waveforms were sampled in real time at rest and during exercise at 1,000 Hz through a Doppler audio transformer (Herr et al., 2010) that connected the Doppler to a data acquisition system (Powerlab 8SP, ADInstruments, Colorado Springs, CO). Blood velocity and diameter were analyzed offline. Mean blood velocity was derived from averaging across cardiac cycles for 30s at rest and over each duty cycle (contraction and relaxation phase) during exercise. Only blood velocity envelopes that were clear and fully captured within the duty cycle were recorded. The average (±SE) number of duty cycles captured across exercise trials was 91 ± 2% and 97 ± 1% for the follicular and luteal groups, respectively, and did not significantly differ between groups (*p* = .14). Average diameter was measured using an automated edge detection system (Brachial Analyzer, Medical Imaging Applications, Coralville, IA) from at least a 20s video clips recorded at rest and during exercise. Forearm blood flow (FBF) was calculated by multiplying the cross‐sectional area (πr^2^) of the brachial artery with mean blood velocity.

Forearm vascular conductance (FVC) was calculated by dividing FBF by mean arterial pressure then multiplying by 100 to express FVC as milliliters per minute per 100 mmHg. Mean arterial blood pressure was measured continuously at rest and exercise using a finger plethysmograph (CNAP monitor 500at, CNSystems, Austria) that involved a double finger sensor that was placed on a motionless left hand resting at the level of the heart.

### 2.8 Blood analysis

Blood was collected anaerobically by venipuncture from an antecubital vein into tubes (BD Vacutainer, Franklin Lakes, NJ) containing K2 EDTA (10.8 mg). Immediately upon obtaining the blood sample, tubes were centrifuged at 2,000 x*g* for 10 min. Subsequently, the supernatant was collected and immediately frozen at −80°C until batch analysis for plasma ARG, estradiol, and progesterone. Due to difficulty inserting the needle into a vein, blood was not collected in all participants for all visits. Thirteen women (eight in follicular, five in luteal) had blood samples for each visit, while eight women (three in follicular, five in luteal) had blood samples from a single visit.

Estradiol and progesterone were measured in duplicate using commercially available enzyme‐linked immunosorbent assays (ALPCO, catalog# 11‐ESTHU‐E01 and 11‐PROHU‐E01, Salem, NH). L‐arginine was measured in duplicate using liquid chromatography‐tandem mass spectrometry (LC‐MS) following extraction methods described by Shin, Fung, Mohan, and Fung (2011) with slight modifications based on available resources. Briefly, plasma samples were spiked with deuterated CIT (D7‐CIT) as a surrogate then treated with LC‐MS grade acetonitrile (ACN) for protein precipitation. Samples were cooled to −20°C for 15min then centrifuged at 16,900*g* for 10 min. A portion of the supernatant was collected for analysis. LC‐MS calibration standards of ARG (Sigma‐Aldrich, St. Louis, MO) were constructed in ACN:H2O (5:1). Calibration standards also contained D7‐CIT at a concentration equivalent to the surrogate concentration in plasma samples, which was used to adjust ARG concentrations for extraction efficiency. Chromatographic separation was carried out using an Ultimate 3000 UHPLC system with quantification by an Endura triple stage quadrupole (TSQ) mass spectrometer (Thermo Scientific, Waltham, MA). The analytical column was a Zorbax Eclipse‐C8 (4.6 × 150 mm, 5 µm). Mobile phase solvents were 0.1% formic acid in water (A) and 0.1% formic acid in 50/50 acetonitrile/methanol (B). Solvent composition (A:B; v/v) was 70:30 at 0–2 min, 30:70 at 4–6 min, 0:100 at 7 min, and 70:30 at 8–10 min at a flow rate of 0.40 ml/min. The injection volume was 20 µl and the column temperature was set to 30°C. Mass spectrometry parameters were optimized for ARG and D7‐CIT prior to sample analysis. We used multiple reaction monitoring in the positive mode for analyte quantitation.

### 2.9 Data analysis and statistics

Resting hemodynamics, including FBF and FVC, was derived from averaging across cardiac cycles for 30s at rest, while exercise hemodynamics was an average from the second minute to fourth minute of exercise. Vasodilator kinetics at exercise‐onset was analyzed using a single‐component model [ΔFBF or ΔFVC = G_0_ + G_1_(1 – e^‐(t – TD)/τ)^], with ΔFBF or ΔFVC representing the duty cycle‐dependent change in blood flow or vascular conductance from baseline (G_0_), G_1_ represents the steady‐state amplitude, TD represents the time delay, and represents the time constant of the response or the time required to achieve 63% of the steady‐state amplitude known as mean response time. We also report the number of duty cycles to achieve 63% of the steady‐state amplitude for FBF (τΔFBF) and FVC (τΔFVC) similar to previous work (Casey et al., 2015). The ΔFBF and ΔFVC (duty cycle – rest) were used for modeling to remove day‐to‐day fluctuation in basal hemodynamics, thus providing a stable baseline (equal to 0) for modeling as described by others (Kellawan et al., 2017).

Independent sample *t*‐tests were used to compare demographic data between women tested during the follicular phase and luteal phase. One‐way analysis of variance was used to assess plasma ARG levels between placebo and CIT conditions within each menstrual cycle group. Two‐way repeated measures analysis of variance (RMANOVA) was used to compare menstrual cycle groups (follicular versus luteal) across treatments (placebo versus CIT). All hemodynamic variables used in the RMANOVA passed normality (Shapiro–Wilk) and equal variance (Brown–Forsythe) tests. Statistical significance was considered *p* ≤ .05.

## 3 RESULTS

### 3.1 Participant characteristics

Table 1 shows participant characteristics for women tested during the follicular and luteal phases. Women were similar in age, body mass, resting seated systolic blood pressure, fasting blood glucose, maximal grip strength, estimated fitness level, and plasma levels of estradiol. Women in the luteal phase group had a lower resting seated diastolic blood pressure (*p* = .03) and higher plasma levels of progesterone (*p* < .01) as compared to women in the follicular phase group.

**Table 1:** Participant characteristics

	Follicular (*n* = 13)	Luteal (*n* = 11)	*p*‐value
Age (y)	24 ± 2	25 ± 2	.55
Weight (kg)	62 ± 9	62 ± 11	.89
Body mass index (kg/m^2^)	23 ± 2	23 ± 2	.91
Seated systolic BP (mm Hg)	112 ± 11	110 ± 7	.55
Seated diastolic BP (mm Hg)	77 ± 7	70 ± 6	.03
Fasting glucose (mg/dl)	95 ± 8	90 ± 14	.25
Maximal grip strength (kg)	28 ± 5	28 ± 4	.96
Estimated VO_2max_ (ml/kg/min)	33 ± 5	35 ± 2	.36
Estradiol (pg/ml)^#^	94 ± 47	80 ± 56	.53
Progesterone (ng/ml)^#^	7 ± 6	28 ± 6	<.01

Note

Values are mean ± *SD*. BP, blood pressure; VO_2max_, maximal oxygen uptake.

^#^ *n* = 11 in follicular group and *n* = 10 for the luteal group.

### 3.2 Change in L‐arginine

**Figure 2:**
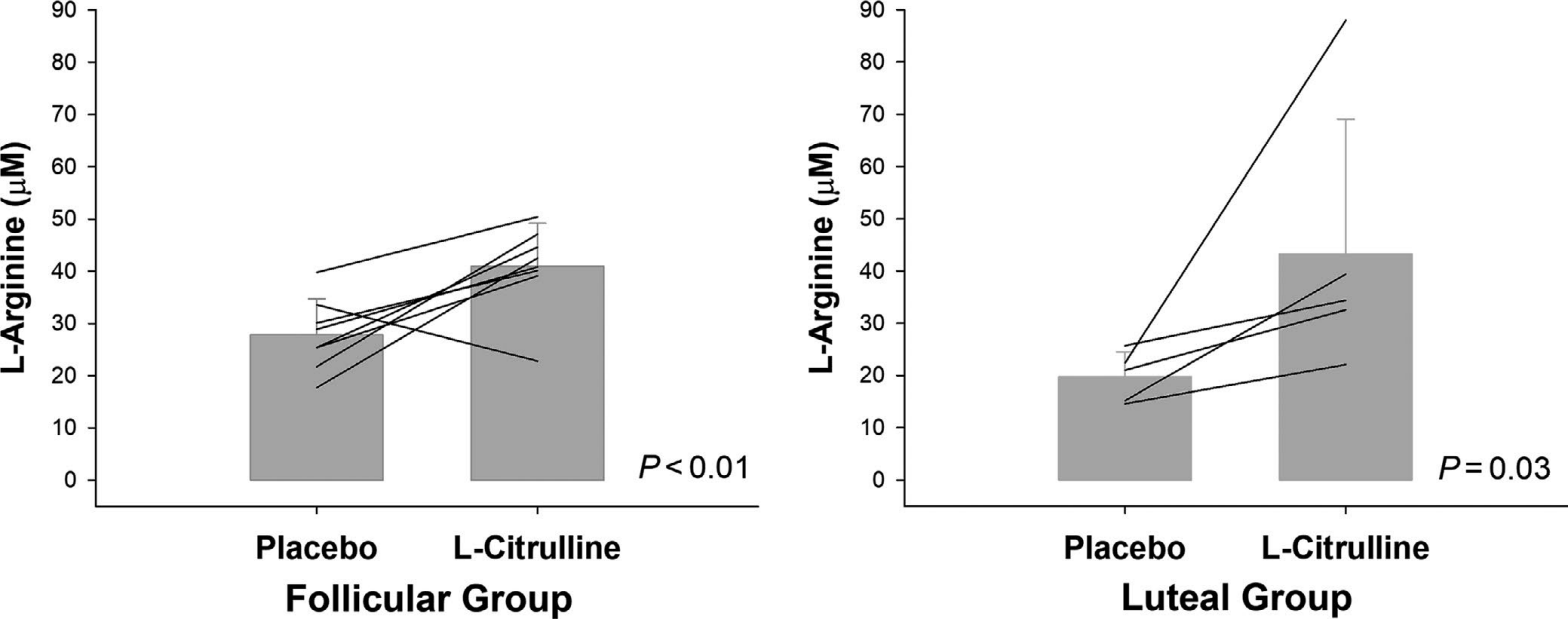
Change in plasma levels of L‐arginine following placebo and L‐citrulline supplementation (6g/day for 7 days) in women tested during the follicular (*n* = 8) and luteal phases (*n* = 5) of the menstrual cycle. Only women with a complete set of blood samples are presented. Data in bar graphs presented as mean ± *SD*

Figure 2 shows the change in plasma levels of ARG in a subset of women when supplemented with CIT during the follicular and luteal phases of the menstrual cycle. L‐arginine was higher in plasma after CIT supplementation as compared to placebo in women tested during the follicular (*p* = .004) and luteal phases (*p* = .03 or *p* = .02 if excluding outlier). The absolute change in plasma levels of ARG following CIT supplementation was not different between women tested during the follicular (Δ13 ± 11 µM) and luteal phase (Δ23 ± 24 or Δ13 ± 7 µM if excluding outlier; *p* = .31 and *p* = .98, respectively).

### 3.3 Resting and exercise hemodynamics

Table 2 compares hemodynamics between placebo and CIT for women tested in the follicular and luteal phases. Resting mean arterial pressure was lower (*p* < .01) in women tested during the luteal phase as compared to women in the follicular phase. As a result, exercise mean arterial pressure (*p* = .055) and heart rate (*p* = .054) tended to be lower in the luteal phase group. Average resting and exercise FBF and FVC including the amplitude of rise in ΔFBF and ΔFVC from rest to steady‐state exercise were similar between menstrual cycle groups and treatment conditions with no interaction found for any variable (all *p* > .05).

**Table 2:** Average resting and exercise hemodynamics

	Follicular		Luteal		Main Effect *p*‐value
	Placebo	L‐Citrulline	Placebo	L‐Citrulline	Group* Treatment**
Heart rate (bpm)					
Rest	71 ± 7	74 ± 11	70 ± 7	68 ± 10	.26 .57
Exercise	80 ± 10	83 ± 12	74 ± 10	72 ± 9	.055 .56
Mean arterial pressure (mm Hg)					
Rest	87 ± 9	90 ± 7	80 ± 7	81 ± 8	<.01 .35
Exercise	97 ± 14	92 ± 17	83 ± 9	87 ± 7	.054 .81
Diameter (mm)					
Rest	3.01 ± 0.36	3.06 ± 0.37	3.21 ± 0.30	3.21 ± 0.25	.18 .50
Exercise	3.37 ± 0.43	3.43 ± 0.47	3.52 ± 0.29	3.45 ± 0.26	.59 .91
Mean blood velocity (cm/s)					
Rest	7.5 ± 2.7	7.9 ± 2.6	6.1 ± 2.1	6.3 ± 2.7	.11 .58
Exercise	30.3 ± 6.1	30.1 ± 4.6	30.0 ± 8.1	29.3 ± 6.4	.80 .71
Forearm blood flow (ml/min)					
Rest	33 ± 17	37 ± 19	30 ± 12	31 ± 17	.47 .41
Exercise	163 ± 47	169 ± 52	175 ± 50	164 ± 40	.86 .63
ΔFBF or Amplitude	124 ± 39	129 ± 39	142 ± 13	131 ± 11	.50 .64
Mean response time (s)	18 ± 9	23 ± 15	29 ± 13	35 ± 16	.02 .10
FVC (ml/min/100 mmHg)					
Rest	38 ± 18	41 ± 20	37 ± 15	38 ± 17	.76 .66
Exercise	170 ± 51	185 ± 51	213 ± 74	194 ± 50	.23 .82
ΔFVC or Amplitude	129 ± 40	136 ± 37	172 ± 19	150 ± 15	.11 .45
Mean response time (s)	15 ± 9	18 ± 10	25 ± 12	28 ± 16	.02 .35

Note

Values are mean ± *SD*. FBF, forearm blood flow; FVC, forearm vascular conductance.

* Main effect for the follicular versus luteal group comparison.

** Main effect for the placebo versus L‐citrulline treatment comparison. There were no group by treatment interactions (*p* > .05).

### 3.4 Vasodilator kinetics during dynamic handgrip

**Figure 3:**
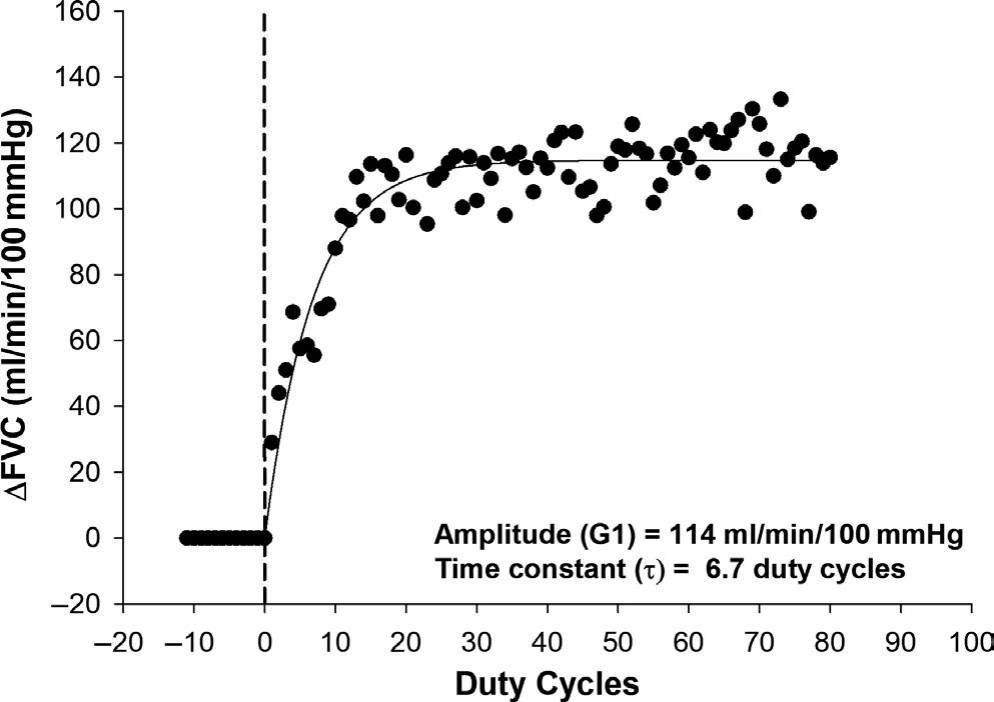
Typical change in forearm vascular conductance (FVC) in response to dynamic handgrip exercise (20 contractions per minute) performed at 10% of maximal voluntary contraction force for a woman tested in the follicular phase after placebo. Dashed vertical line indicates exercise onset and the solid curve line is the monoexponential fit

**Figure 4:**
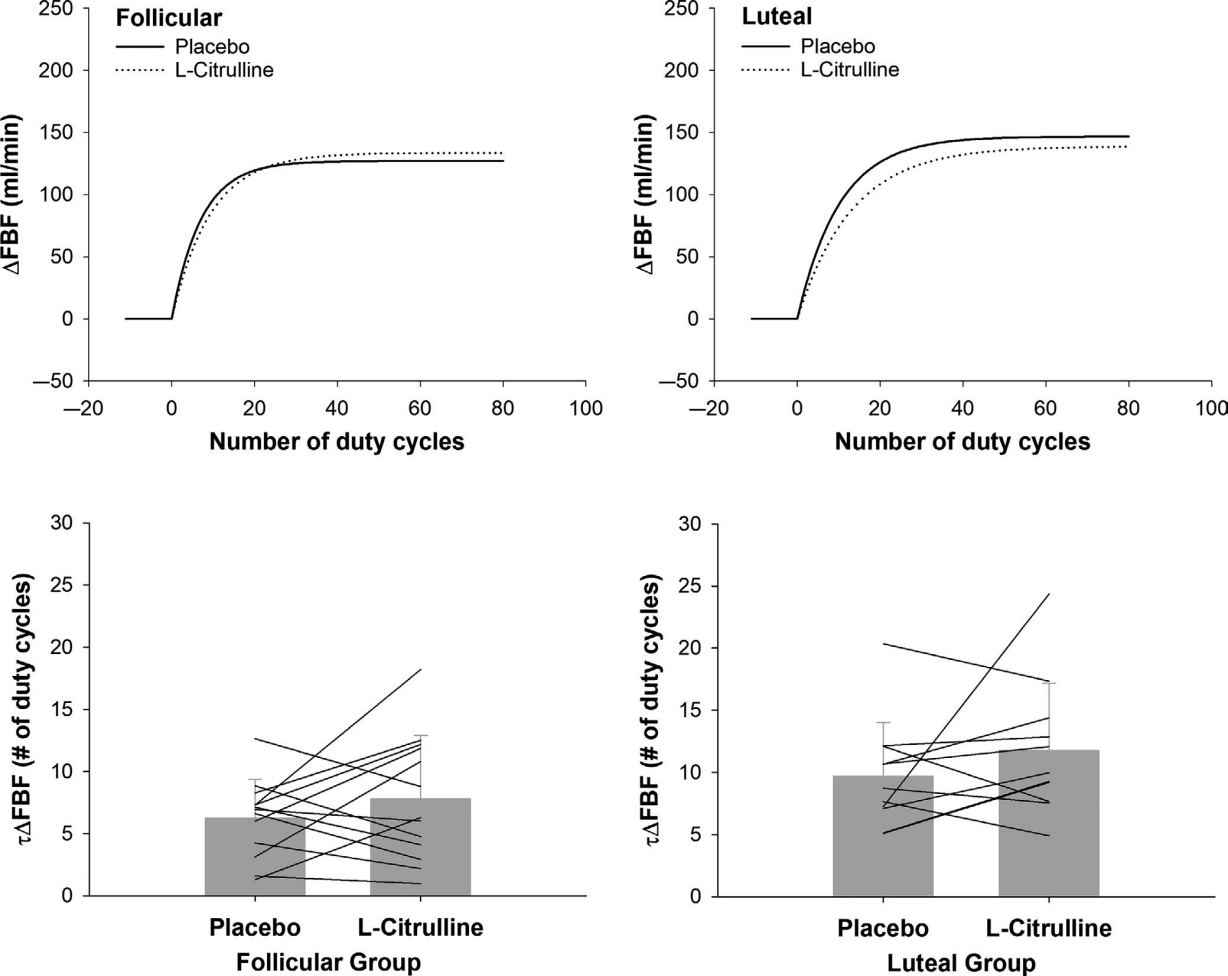
*Upper panels*: Mean fit for the on‐transient forearm blood flow (FBF) kinetics during dynamic handgrip exercise in young women following placebo and L‐citrulline supplementation. *Lower panels*: Comparison of the number of duty cycles to reach 63% of the steady‐state amplitude forearm blood flow (τΔFBF) during dynamic handgrip exercise between placebo and L‐citrulline conditions in women tested during the follicular and luteal phases of the menstrual cycle. Bar graphs are mean ± *SD*

**Figure 5:**
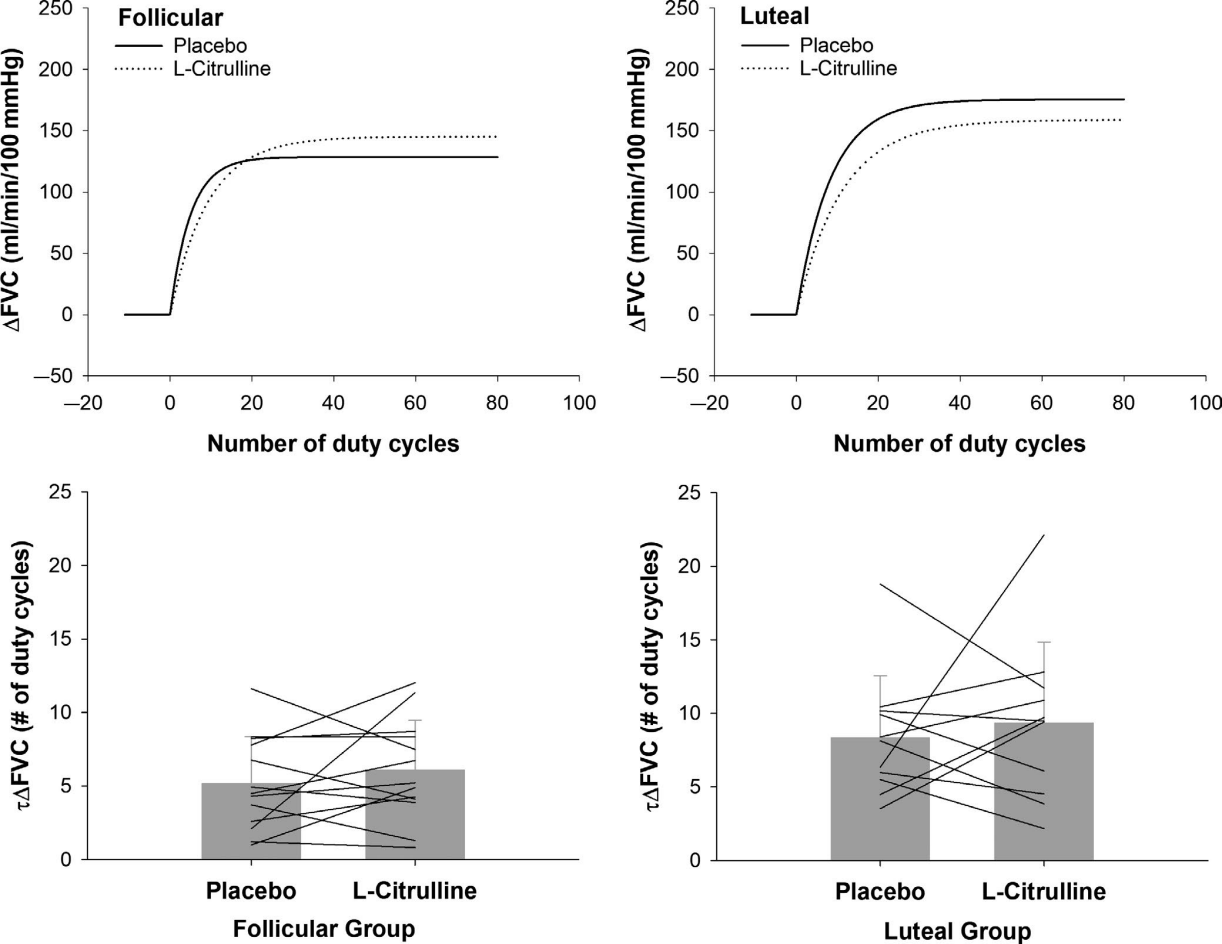
*Upper* panels: Mean fit for the on‐transient vasodilator kinetics (forearm vascular conductance; FVC) during dynamic handgrip exercise in young women following placebo and L‐citrulline supplementation. *Lower panels*: Comparison of the number of duty cycles to reach 63% of the steady‐state amplitude forearm vascular conductance (τΔFVC) during dynamic handgrip exercise between placebo and L‐citrulline conditions in women tested during the follicular and luteal phases of the menstrual cycle. Bar graphs are mean ± *SD*

Figure 3 shows an example tracing of the monoexponential modeling of raw data from a representative participant in the luteal phase group after taking placebo. No effect of CIT was observed for τΔFBF (Figure 4, treatment effect, *p* = .11) or τΔFVC (Figure 5, treatment effect, *p* = .35) such that the number of duty cycles for FBF and FVC to reach steady‐state amplitude was not different following CIT supplementation as compared to placebo in women tested during the follicular or luteal phase. However, a main effect for group (*p* = .02) was observed for the number of duty cycles for FBF and FVC to reach steady‐state amplitude suggesting slower kinetics in women tested during the luteal phase as compared to women tested during the follicular phase (τΔFBF: 10 ± 1 versus 7 ± 1 duty cycles; τΔFVC: 8 ± 1 versus 5 ± 1duty cycles). These findings are consistent with those for mean response time reported in Table 2.

## 4 DISCUSSION

In this study, we aimed to determine whether CIT supplementation improves vasodilator kinetics at the onset of dynamic handgrip exercise in healthy, young women supplemented during the follicular or luteal phases of the normal menstrual cycle. We hypothesized that CIT supplementation would have a differential effect on vasodilator kinetics relative to menstrual cycle phase. Although we were able to show for the first time that CIT supplementation increases plasma levels of ARG in young women, we found no effect of CIT supplementation on the hyperemic and vasodilatory responses to exercise irrespective of menstrual cycle phase.

The importance of the research question addressed in this study is translational relevance as work in cell culture indicates hormonal influences on the ability of ARG to be transported into the endothelial cell for nitric oxide production. Bentur et al. (2015) has shown that administration of estradiol to cultured endothelial cells increased uptake of ARG while co‐incubation with progesterone significantly reduced ARG transport into the endothelial cell. This hormonal influence was due to modulation of phosphorylated ERK and PKCɑ levels that impacted activity of the endothelial transporter CAT‐1. As CIT supplementation significantly increases plasma levels of ARG (Bailey et al., 2015; Kim et al., 2015; Moinard et al., 2008; Schwedhelm et al., 2008), as confirmed in young women in this study, we hypothesized that vasodilator kinetics would be improved when CIT supplementation occurred during the follicular phase when estradiol is elevated and progesterone is low. However, our results showed no effect of CIT supplementation on ΔFBF and ΔFVC responses to exercise in women tested during the follicular phase suggesting that this menstrual cycle phase is not important for supplemental ARG to enhance nitric oxide production in healthy young women.

Additionally, we observed no effect of CIT supplementation in women tested during the luteal phase. Women in this phase had significantly higher plasma progesterone levels than women tested during the follicular phase (Table 1). We expected that CIT supplementation would be ineffective at improving vasodilator kinetics at the onset of exercise in women tested during the luteal phase due to progesterone's reported role of decreasing endothelial CAT‐1 activity (Bentur et al., 2015). While our results may seem consistent with this hypothesis, the absence of improved ΔFBF or ΔFVC responses to exercise in women tested during the follicular phase (when progesterone levels are low), suggests progesterone had no relevance on whether CIT supplementation had a positive effect on endothelial nitric oxide production in healthy young women.

An interesting observation made by this study is that progesterone level did not negatively influence the ability of CIT supplementation to augment plasma ARG level as evidenced by similar absolute increases in plasma ARG following CIT supplementation between women tested during the follicular phase when progesterone was low, and the luteal phase when progesterone was elevated. This finding indicates that the often reported decrease in plasma ARG in the luteal phase of the menstrual cycle (Faustmann et al., 2018; Valtonen et al., 2010) is not likely due to the ability of the kidney to convert CIT into ARG by the enzymes argininosuccinate synthase and argininosuccinate lyase in a partial urea cycle. Therefore, CIT should be considered as a therapeutic option for women that may have deficiencies in ARG availability such as conditions associated with elevated arginase activity like obesity (Johnson et al., 2015).

Vasodilator kinetics at the onset of handgrip exercise measured in this study was similar to those reported in previous work. Casey et al. (2015) reported ~ 7 duty cycles to reach 63% of steady‐state FVC for young men exercising at 10% maximal grip strength during dynamic handgrip exercise. The combined average (placebo + CIT conditions) in the present study was 5 and 8 duty cycles for FVC to reach steady‐state in women tested during the follicular and luteal phases, respectively. This comparison suggests exercise‐onset vasodilatory kinetics during handgrip exercise may be similar between young women and men, although it is possible that vasodilatory kinetics may be faster in women tested during the follicular phase. In the present study, both mean response time and number of duty cycles to reach 63% of steady‐state FVC was faster in women tested during the follicular phase than women tested during the luteal phase (*p* = .02 for group comparison). However, this result should be interpreted with caution as we did not examine the same women across the follicular and luteal phases. Thus, the slower vasodilatory kinetics in women tested during the luteal phase in this study may be due to inter‐individual variability and not related to menstrual cycle phase. Nevertheless, this observation is interesting and worthy of attention in future research using a study design that examines the same women across each menstrual cycle phase.

Few studies have compared the effectiveness of CIT supplementation on vascular function in women alone or separately from men. Figueroa, Wong, Hooshmand, and Sanchez‐Gonzalez (2013) found that CIT supplementation improves vascular function as reflected by lower resting peripheral and central blood pressure along with reduced arterial stiffness (Figueroa et al., 2015) in obese postmenopausal women with hypertension. However, this is in contrast to other work that finds no effect of CIT supplementation on resting blood pressure, central arterial stiffness, or femoral blood flow during leg exercise in healthy nonobese older women (Gonzales et al., 2017). Moreover, Ashley et al. (2018) showed no effect of CIT supplementation on pulmonary oxygen uptake kinetics at the onset of walking in a combined group of healthy young and older women. Our results add to the literature that does not support CIT supplementation having a positive impact on vascular function in healthy women despite significantly increased plasma levels of ARG. This is in contrast to young men, where 7‐days of CIT taken at 6 g/day has been reported to lower resting blood pressure and improve skeletal muscle oxygenation patterns during cycling exercise (Bailey et al., 2015). Collectively, the findings mentioned above highlight an emerging key point regarding the efficacy of short‐term CIT supplementation in that this amino acid may only improve vascular function in women that have pronounced vascular dysfunction (e.g., obesity, hypertension).

Several limitations accompany this study. First, we did not assess women during the early follicular phase when circulating estradiol and progesterone levels are low. Including this phase would have allowed for a comparison of the effect of CIT supplementation on vasodilator kinetics between conditions of low and elevated estradiol levels. Second, as mentioned above, we did not assess the same women across different phases of their menstrual cycle in order to shorten the study duration for participants since washout periods were required between placebo and CIT treatments. This prevents us from making concrete conclusions from our comparison of vasodilator kinetics between menstrual cycle phases. Lastly, D7‐CIT was used as a surrogate/internal standard in our LC‐MS measurements of plasma ARG (Shin et al., 2011). Without a stable isotope of ARG for an internal standard, we were unable to account for ionization suppression which is common for mass detectors. This is likely the cause of our relatively low values of ARG in comparison to the literature (Faustmann et al., 2018; Valtonen et al., 2010), but we assumed ionization suppression did not vary among samples; thus, the underestimation should be consistent across all samples.

In conclusion, 7 days of CIT supplementation at 6 g/day does not alter forearm blood flow and vascular conductance responses to dynamic handgrip exercise in young, healthy women despite increasing plasma ARG concentrations. Furthermore, menstrual phase did not impact CIT effects on exercise hemodynamics. Therefore, using CIT to potentiate the ARG‐nitric oxide pathway does not improve vasodilator kinetics in young, healthy women.

## CONFLICT OF INTEREST

No conflicts of interest, financial or otherwise, are declared by the authors.

## AUTHOR CONTRIBUTIONS

This study was conducted in the Department of Kinesiology and Sport Management at Texas Tech University. All persons designated as authors qualify for authorship, and all those who qualify for authorship are listed. JUG and AF conceptualized and designed the study. JUG, SF, and AM collected the data. AF randomized the order of treatments and blinded the researchers until data collection and analysis was complete. HV analyzed blood samples for plasma hormone levels. TA, AK and SS determined L‐arginine concentrations in plasma samples. JMK assisted with the vasodilator kinetic analysis. All authors provided feedback on the manuscript text and approved the final version.
